# Identifying population segments for effective intervention design and targeting using unsupervised machine learning: an end-to-end guide

**DOI:** 10.12688/gatesopenres.13029.2

**Published:** 2019-10-21

**Authors:** Elisabeth Engl, Peter Smittenaar, Sema K. Sgaier

**Affiliations:** 1 Surgo Foundation, Washington, DC, 20011, USA; 2 Department of Global Health & Population, Harvard T.H. Chan School of Public Health, Boston, MA, USA; 3 Department of Global Health, University of Washington, Seattle, Washington, USA

**Keywords:** Psycho-behavioral segmentation, human heterogeneity, global health and development; targeted intervention design, behavioral science, cluster analysis, unsupervised learning, machine learning

## Abstract

One-size-fits-all interventions that aim to change behavior are a missed opportunity to improve human health and well-being, as they do not target the different reasons that drive people’s choices and behaviors. Psycho-behavioral segmentation is an approach to uncover such differences and enable the design of targeted interventions, but is rarely implemented at scale in global development. In part, this may be due to the many choices program designers and data scientists face, and the lack of available guidance through the process. Effective segmentation encompasses conceptualization and selection of the dimensions to segment on, which often requires the design of suitable qualitative and quantitative primary research. The choice of algorithm and its parameters also profoundly shape the resulting output and how useful the results are in the field. Analytical outputs are not self-explanatory and need to be subjectively evaluated and described. Finally, segments can be prioritized and targeted with matching interventions via appropriate channels. Here, we provide an end-to-end overview of all the stages from planning, designing field-based research, analyzing, and implementing a psycho-behavioral segmentation solution. We illustrate the choices and critical steps along the way, and discuss a case study of segmentation for voluntary medical male circumcision that implemented the method described here. Though our examples mostly draw on health interventions in the developing world, the principles in this approach can be used in any context where understanding human heterogeneity in driving behavior change is valuable.

## Introduction

People are not all the same. For example, a public health program might want to understand the different reasons that drive young women at risk of HIV infection to start or stay away from pre-exposure prophylaxis ^1– 3^. Another program might want to convince households to reduce the use of coal for cooking fires. In the private sector, a weight loss company might plan to deliver targeted messages to dieters to help them stick to their goals. In each case, the particular behavior of interest may be shaped by very different drivers, whether they are barriers or enablers ^4^. Simply put, not all customers are motivated in the same way to achieve the same goals.

Segmentation is an approach to uncover these drivers by dividing a population into distinct sub-groups (‘segments’) that each share defining characteristics. Psycho-behavioral segmentation emphasizes that these differences may be due not only to variation in demographic or socio-economic factors, which are perhaps the most basic differentiators traditionally used to form sub-groups, but also to varying behavioral patterns, the underlying drivers of behavior such as beliefs, and other external and perceptual drivers ^5– 7^.

Private-sector market research has long understood the benefits of psycho-behavioral segmentation for persuasion, and in one form or another companies have been applying this method to micro-target customers for several decades ^7, 8^. Segmentation works: in a recent study, matching messages to segment-specific drivers increased online purchase behavior by up to 50% compared to mis- or unmatched messaging ^9^. This approach is also being adopted by the public and development sectors, albeit at a slower pace ^5^.

There are several barriers to scaled adoption of psycho-behavioral segmentation: as well as funding gaps, a likely reason is the lack of expertise, which so far has been very much concentrated within the private market-research sector and pockets of academia. The private sector’s expertise in designing, fielding, and analyzing segmentation research is rarely made transparent for others to replicate or adapt. At the other extreme, the characteristics and assumptions behind the data science of segmentation algorithms have been detailed extensively in the scientific literature ^2, 10^. What is needed is a resource that pulls together both program-level and technical considerations, enabling stakeholders in the development and private sectors to identify and communicate useful segmentation solutions. To our knowledge, there is no end-to-end guide that covers both these program-level and technical aspects of segmentation, a gap this article aims to resolve.

**Figure 1.**
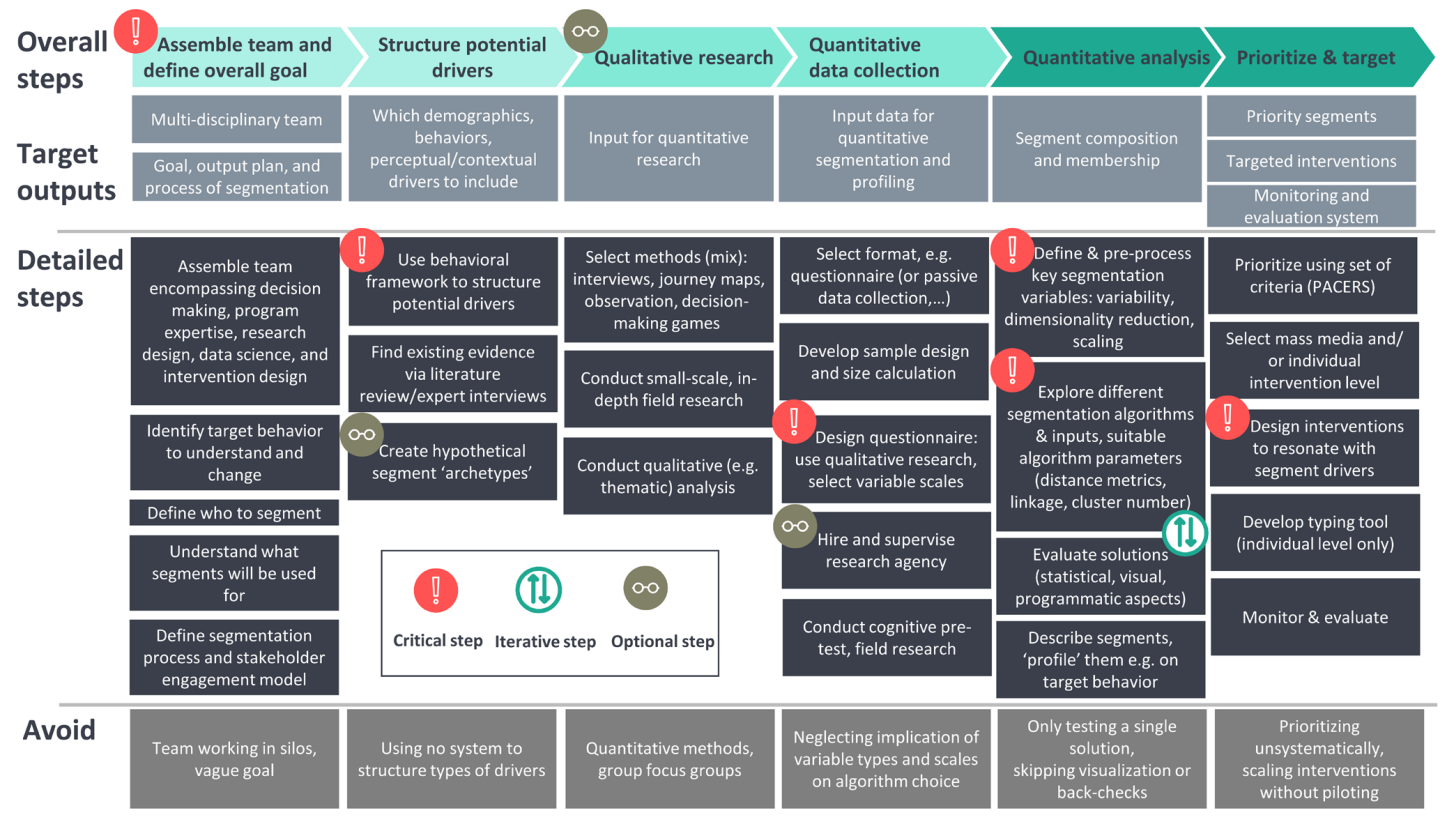
Process flow for end-to-end segmentation. This pathway captures the overall and detailed steps and decision points, as well as target outputs, at each step from conceptualization to research design, implementation, analysis, and targeting. It is meant to assume the maximum workload for a program, where a de novo data set needs to be generated through primary research. Other scenarios are possible. For example, after defining a goal and structuring potential drivers, programs may find they can use existing data sets, in which case they might jump straight to cluster analysis. In any case, critical steps are interlinked and depend strongly on each other. They should therefore be considered together from the start.

Robust and actionable psycho-behavioral segmentation involves many choices, and this article outlines best-practice guidelines for all the steps in the process ( Figure 1). These include assembling the right expertise in a team, determining what group of people to segment, deciding whether and how to design qualitative and quantitative primary research and select appropriate inputs for segmentation, making sound analytical choices in cluster analysis, and assessing how to prioritize segments and identify segment membership at an individual level in the field. Appropriate interventions that are segment-specific, rather than one-size-fits-all, can then be designed and delivered through the right channels.

We aim to make the process of design, analysis, and application of segmentation accessible to all stakeholders involved in a segmentation project. Collaboration is key, and technical and context experts need to ask each other the right questions. For example, a researcher will find it useful to understand what the program ultimately aims to do with the segmentation, or how many segments it will be able to intervene on. Conversely, a program manager can benefit from understanding the analytic choices a data scientist makes (such as which variables to include), as these must reflect the context of the program and will result in different outputs.

So-called ‘unsupervised’ machine-learning algorithms that include cluster analysis methods are a primary analytical tool and a focus of this article. Throughout the process of segmentation, iteration is crucial, and segmentation is both a science and an art. Practitioners have to consider what the segmentation will ultimately be used for, choose which data and variables to use, experiment with several analytical techniques, and check results against expert knowledge and the real-world value of the results.

The steps described here are comprehensive, and we expect researchers and implementers to adapt, skip, and expand various parts of this approach depending on their own situation. Some programs may already have data, and we encourage them to consider its value for segmentation before starting from scratch. In other cases, expert domain knowledge may enable programs to skip undertaking secondary or exploratory primary research. What both researchers and implementers should take away, however, is that understanding and efficiently changing behavior implies understanding differences not only in demographics and patterns of behavior, but also in the values and beliefs underlying these behaviors. This article outlines the principles of uncovering and addressing this heterogeneity, and we discuss them in light of the choices made in a recent study on voluntary medical male circumcision for HIV prevention in Zimbabwe and Zambia ^3^.

## Methods

### Assembling key stakeholders and defining overall goals

Technically sound and actionable segmentation depends on the contributions of a multi-disciplinary team, encompassing a set of distinct characteristics: overall decision-makers, program domain experts, research designers with behavioral expertise, data scientists skilled at cluster analysis, and intervention designers. A good solution across all steps involved in segmentation ( Figure 1) depends on collaboration and mutual pressure-testing.

Once the team is assembled, it is critical to clarify and align on the ultimate aim of the segmentation project: What is the target behavior for which differences in drivers such as beliefs, structural constraints, or demographics should be understood? Who should be segmented? And what could segmentation ultimately be used for? For example, a public health program may want to reduce the impact of malaria in a region. The primary target behavior might be the use of bed nets, or care-seeking behavior once first symptoms are detected, or medication adherence. Each of these behaviors will have different drivers that serve as input variables for cluster analysis. They will also encompass different key stakeholders, such as existing malaria patients or mothers making purchasing decisions for their households. People might also be segmented for different purposes, for example to be individually targeted by active case finding teams, or alternatively so that governments can offer a portfolio of products that might appeal to different segments. Being clear about who to segment and for what purpose also allows researchers to later narrow down the potential input variables more efficiently, and to create meaningful and actionable segments.

### Structuring potential variables as inputs for segmentation

Segmentation requires quantitative data – e.g. numbers, scores, scales, categories – containing a range of variables that are likely to be related to the target behavior, that vary among people, and at least some of which are amenable to change via interventions. The algorithms will rely solely on these variables to group people together. Choosing too many variables will lead to segments poorly differentiated on each individual variable. Segmenting on variables that are not related to a relevant target behavior will not enable programs to design interventions that change outcomes of interest. A common pitfall is that the segmentation algorithm will always find *some* segmentation, no matter how poorly the segments truly differentiate on the underlying variables or how unactionable the solution is. It is up to the practitioners to make sure the inputs and outputs make sense, and an expert understanding of the problem, people, and context should inform what variables are used as input. Most often, this requires conducting primary research.

Brainstorming an initial list of variables is critical, including demographic characteristics, behaviors, and external and perceptual barriers and enablers related to the target behavior ^4^. Erring on the side of listing an exhaustive number of variables can be useful: not all will serve as inputs to the final segmentation, but missing key factors associated with a target behavior will greatly compromise the value of the final output.

***Categories of variables: demographic, behavioral, perceptual, contextual.*** A comprehensive range of variables describing demographic characteristics can be captured, including age, location, income, education level, and other socioeconomic characteristics. Most of these variables will not be used to make up psycho-behavioral segments. Instead, they may later be used to ‘profile’ segments – the process of describing segments based on variables not included in the segmentation itself – and narrow down where interventions should be targeted.

For variables capturing behaviors, it helps to focus on those that are likely related to the target behavior. For example, if patients are to be segmented on their barriers to seeking medical care, data collection may aim to capture their previous interactions with healthcare providers, general self-care behaviors, and information-seeking.

A well-validated framework of behavioral theory can help structure potential contextual and perceptual drivers. The CUBES (to Change behavior, Understand Barriers, Enablers, and Stages of change) behavioral framework, for example, can be used to help programs list the types of drivers that could potentially be involved in any given behavior, evaluate existing evidence on what drivers actually are important, and design primary research around closing evidence gaps ^4^. CUBES was synthesized from the strongest evidence-based approaches to systematically investigating behavior ^9, 11, 12^. It posits that behavior occurs on a continuum from awareness to intention, action and beyond, and various factors can enable or obstruct movement along that spectrum. These drivers can be contextual – such as infrastructure, regulations, or systems and processes – or perceptual, such as a person’s beliefs, emotions, and biases. Layers of influencers and channels mediate these drivers.

***Utilizing existing research to identify knowledge gaps.*** Using the structure of potential drivers identified above, a structured literature review and/or expert interviews can be conducted to find what is already known about likely barriers and enablers of the target behavior. For example, a study may find that beliefs about the health risks of a procedure are a key barrier to patients electing to undergo it. For purposes of segmentation, it is useful to focus on the barriers and facilitators that do not show widespread agreement in the target population, as these have the potential to create distinct segments. The findings can then be used to update the list of variables for upcoming data collection and make them more specific. For example, while a framework of behavior might provide a pointer to look for risk perception beliefs, secondary research can help refine exactly what risks people worry about.

Based on the literature review and/or expert interviews, a list of ‘evidence blind spots’ can then be compiled: which behaviors or drivers of behaviors are well understood, and which ones are not? For example, is there plenty of evidence on socioeconomic factors and infrastructure driving the target behavior, but nothing on social norms? If there is enough information to design structured quantitative research, qualitative research (see below) may be skipped.

At this stage, programs may find it helpful to form initial hypotheses on how potential drivers could group together, and sketch out tentative segments, or archetypes, bringing presumed essential differences to life. Archetypes can provide a clear vision of what needs to be pressure-tested qualitatively and/or quantitatively, and can be refined again after qualitative research. However, it is also important to remain open-minded that the segments resulting from the data may differ from the hypothesized groupings.

### Exploratory qualitative research

When relatively little is known about a target behavior, qualitative primary research is an excellent way for programs to deepen their knowledge in an exploratory way and ultimately improve their chances of a successful segmentation. For segmentation, the goal of qualitative research is to help structure and refine quantitative research items. This step can therefore be skipped if there is extensive existing knowledge of the likely drivers of a target behavior. If programs decide to design and conduct qualitative research, they can select a mix of qualitative methods to investigate the identified blind spots in the target population in depth, balancing their relative strength and weaknesses. Examples include in-depth interviews, ‘journey mapping’, which tracks behaviors and attitudes over time, decision-making exercises, and observations ^3, 4^. Quantitative surveys are not suitable at this stage, as the aim is to allow for open responses and unexpected insights. Focus groups, which may obscure individual differences as individual respondents interact with each other, may also be less useful to investigate heterogeneity.

To implement qualitative research, programs can hire a reputable research agency for field implementation of the qualitative methods and supervise training of research staff. A small sample, perhaps 20–30 respondents, is often sufficient, as the goal at this stage is depth of insight to refine further research, not representativeness. For analysis of qualitative research, programs can take advantage of standard qualitative approaches such as thematic analysis, so that patterns (‘themes’) in the data can be identified ^13^. Themes can correspond to the list of input variables defined previously, in which case responses can provide insight into how questions should be phrased for quantitative research, or what answer options should be provided. The qualitative research can also yield new themes and input variables.

### Quantitative data collection

Segmentation requires quantitative data – often from questionnaires – to identify subgroups. Some programs will already have collected or obtained a data set that they want to perform segmentation on. If these data meet the requirements for quantitative segmentation analysis (see below), such as containing relevant variables for segmentation, a large enough sample size, relevant profiling variables, and response scales that allow for sufficient variability, a program might jump to quantitative analysis and not collect any new data at all. For those that do not yet have data or consider their existing data unsuitable for segmentation based on these parameters, this section explains how to optimize quantitative data collection for the purpose of segmentation.

***Defining the scope and sampling approach.*** Defining the scope and size of quantitative research requires balancing the desire for nuanced information with available resources. As with any quantitative research, larger samples can generate a more reliable picture of the population. In addition, the larger the sample, the greater the number of variables that can be included to segment on, and the more nuanced the segments that can be identified. One modeling approach suggests that 100 observations per variable is a good rule of thumb ^14^, though generally the need for observations grows exponentially with number of input variables. Therefore, a robust quantitative segmentation design will usually include at least several hundred and perhaps over a thousand respondents. On the other hand, if programs already know they are limited to interventions that act on only a small set of drivers (and, in turn, few variables are needed to segment on), and resources for data collection are scarce, more focused segmentation can be done on a smaller scale.

For the sample design, researchers can factor in success and failure rates of the target behavior: for example, to understand barriers to using bed nets for malaria, sampling units should not primarily consist of populations with a very high saturation of bed net use.

### Questionnaire design

Reference can be made to strong themes emerging from qualitative research outputs to decide what questions to ask and how, following best practice to capture behavioral drivers. For example, if people are to be asked about their risk perceptions and self-efficacy in relation to disease prevention, existing published question items can be adapted ^15^. Table 1 shows an example of how different drivers used for segmentation and profiling were converted to survey items for a recently-published segmentation study on the drivers of voluntary male medical circumcision in Zambia and Zimbabwe, discussed in the Results section ^3^.

**Table 1.** Converting behavioral drivers into survey items for data collection. These sample items, linked to distinct drivers of behavior ^4^, are an excerpt from a questionnaire used to survey Zimbabwean men aged 15–29 to investigate how men relate to voluntary male medical circumcision ^3^. VMMC - Voluntary medical male circumcision.

Construct / behavioral driver	Scale	Sample item
Sample variables used as segmentation inputs		
**Accurate awareness of** **VMMC**	**7-point Likert scale** (strongly disagree – strongly agree)	Once circumcised, a man cannot get any disease, so no longer has to use condoms to prevent HIV
**Outcome expectation** **beliefs - cost/benefit**	**5-point Likert scale** (definitely no benefits – definitely are benefits)	Based on what you know about circumcision, which of the following statements best describes what you believe about any benefits that male circumcision may give you?
**Risk perception beliefs** **- severity**	**7-point Likert scale** (strongly disagree – strongly agree)	HIV/AIDS is a big problem in our country/community/for people I know/my family
		During the healing process after male circumcision, one experiences constant pain which is difficult to manage
**Risk perception beliefs** **- susceptibility**		I believe that chances are high that I could get HIV
**Self-efficacy beliefs**		It is difficult to control whether you get HIV or not - even if I do my best I still can get it
**Social norms**	**5-point Likert scale** (don’t talk to any other men – bring up the subject even with men I don’t know)	Which of the following statements best describes your discussions with other men about male circumcision?
Sample variables used for profiling		
**Influencers**	**Categorical**	Who or which sources of information have encouraged you to start to believe in benefits of male circumcision?
**Demographics**	**Categorical**	What is your relationship status?
**Circumcision status**	**Categorical**	Are you circumcised?
**Commitment to** **circumcision**	**5-point Likert scale** (definitely not – definitely)	Considering what you know about male circumcision, which statement best describes how willing you would be to get circumcised if the service were free for you?
**Media habits**	**Categorical**	How do you normally look for healthcare information on the internet?
**Risk behaviors**	**7-point Likert scale** (strongly disagree – strongly agree)	I use condoms all the time with my current sexual partner(s)

The analysis of likely drivers of the target behavior might suggest that only a few variables are sufficient to explain differences in the target behavior in the population. In this case, questionnaires to collect segmentation data can be as short as a few minutes, only asking the critical questions. At the other end of the spectrum, large uncertainty about which drivers are most relevant will require collecting a larger set of variables for exploratory analysis. In any case, primary research should always adhere to best-practice design guidelines, such as avoiding over-long questionnaires to preclude survey fatigue.

Even at the design stage, it pays to plan the variable scales needed for various segmentation algorithms. To use the most straightforward segmentation algorithms, variables used as segmentation inputs are best captured as continuous or approximating-continuous scale (such as Likert scales). Continuous variables can still be transformed into categorical data if necessary, and segmentation algorithms exist to account for a mix of continuous and categorical data (see below). Other variables, such as those used for profiling (describing) segments, can use any scale.

If resources allow, data which may be useful later to profile segments can also be captured. There is no fixed rule on which variables are used as segmentation inputs and which for profiling, but it is advisable to segment on drivers that do not fluctuate frequently and are useful in defining targeted interventions. In contrast, profiling variables provide context and can enrich the program’s understanding of the segments, making it easier to define strategies for identifying and targeting them. For example, a variable of ‘favorite TV shows’ is not a suitable input to segmentation, as it may frequently change and segments would therefore not remain stable over time; but segments can be profiled on which TV shows they currently prefer to enrich the narrative. One advantage of relegating some variables to profiling is that it limits the number of dimensions of segmentation, and therefore helps to sharpen differences along the most relevant dimensions.

Before deploying a questionnaire at scale, it is useful to conduct a ‘cognitive pre-test’ of the research instruments in the field. Respondents can be de-briefed to gauge how a question is interpreted and whether the question and its scale yield useful responses. A question that is misunderstood, or results in little variation among answers, can then be revised or removed.

For data collection, programs can again hire a research agency. Research agencies specializing in quantitative data collection may not be the same as those focusing on qualitative research. When data collection is outsourced, it may be advisable to supervise training of research staff on the study purpose, understanding and delivery of questions, probing, and response coding. While training protocols vary, effective staff training should be both classroom-based, including role-playing exercises, and supervised in the field.

### Quantitative segmentation analysis

Even in a lean data set, analysts are commonly faced with more variables than it is wise to include in a segmentation, and the variables might be in a format not conducive to the algorithms that can be chosen. The optimal segmentation solution will not be known beforehand, and iteration is critical. Analysts and the entire team can include different sets of variables, transform the variables in different ways from continuous to categorical, create composite variables, try different algorithms with different parameters, and critically evaluate solutions to assess whether a particular analysis is worthwhile. The considerations below should therefore be considered as part of an iterative process, rather than a series of steps traversed only once.

***Determining the variables entering segmentation.*** Initial descriptive data exploration is key, and it is often very revealing to simply look at the distribution of each variable to identify those that vary most strongly between people. Variables with low variability may be important predictors of behavior, and so may be targets of an intervention in their own right, but are rarely useful for segmentation. Merely examining histograms for each of the variables often provides ample insight into the population and hones an intuition for the data which are critical for the subsequent multiple iterations of analysis. Similarly, correlations and scatterplots between variables can be examined to understand basic patterns of co-variation.

A questionnaire will almost always include several items testing for the same underlying driver. Entering each of these questions in the segmentation will overweight the importance of that driver in the construction of the segments. The solution is to perform dimensionality reduction, which simply means bringing down the number of variables used as input to the algorithm. Most commonly this happens by combining several answers into a single composite score. For example, several questions testing for knowledge about what a medical commodity is and where it is available can form a composite testing for ‘awareness’. Another option is to hand-pick one of a group of questions that is deemed most relevant and representative of the driver, removing the other variables. Lastly, statistical methods to perform dimensionality reduction such as canonical correlation, principal component analysis, or factor analysis can be used. These can also inform researchers which variables are most representative of the underlying constructs, a helpful indicator in selecting variables.

***Pre-processing: scaling and transforming variables.*** As unsupervised cluster analysis based on measures of dissimilarity between individuals that have vastly different value ranges (e.g. one variable expressed in years and one in days) can overstate the importance of some variables simply because of their scale. Scaling variables, for example as standard deviations from the mean, is often a necessary step. However, if there are categorical, ordinal, or binary variables, scaling can be skipped and alternative ways of calculating distances, such as Gower distance, can be used (see below). Though it is advisable to collect continuous responses where possible (because they tend to be richer in information than categorical responses), there will be cases where most variables in the data were collected as categorical. Excellent segmentation algorithms exist that only take categorical variables (such as latent component analysis), but this will require the remaining continuous variables to be converted to categorical variables by cutting the distribution at sensible points. What makes for a ‘sensible’ cut depends on expert knowledge, the distribution of data, and trial and error to see what yields the most useful segments.

***Exploring competing segmentation algorithms.*** The development of segmentation algorithms has been an active area of research for at least half a century ^16^. There is no universal best algorithm, but rather the optimal algorithm must be discovered through an understanding of the data, strengths and weaknesses of the algorithms themselves, and evaluation of the algorithm’s output against project goals. Fortunately, only a few algorithms need to be understood to satisfactorily solve the vast majority of use cases. We provide a brief overview of several of these methods ( Table 2) and describe in more detail two common unsupervised algorithm families: hierarchical clustering and k-means clustering.

**Table 2.** Algorithm choice is defined by the shape of the underlying input data, weighing advantages and disadvantages. Distance-based clustering consists of relatively simple algorithms and is most widely used. Model-based clustering has many applications, and latent class analysis is particularly worthwhile when only categorical data exist. TwoStep clustering is a model-based method but also relies on estimated distances between individuals. As discussed in the text, supervised clustering using decision trees can be useful in select circumstances. “Manual segmentation” is added to emphasize that often an entire population is divided into subgroups for targeting without the use of any algorithm, e.g. by manually setting an age or a geographic cut-off based on prior knowledge or examination of descriptive statistics. Overview of common cluster analysis algorithms.

Algorithm name	Input data	Particularly useful for	Advantages	Disadvantages	Example
Distance-based unsupervised clustering					
**Hierarchical** **clustering**	Dissimilarity matrix	Quick explorations, visualizing clustering solutions with dendrograms	Versatile technique as it can use any distance metric. Dendrogram gives useful intuition on number of clusters.	Commits to joining most adjacent records without reconsidering groupings – might require e.g. k-means to refine clusters.	Clustering symptoms of non-severe malaria in semi-immune Amazonian patients ^18^
**K-means**	Continuous variables only (standardized)	Continuous data sets, quick exploration of data	Simple, widely used, many tools to evaluate optimal # of clusters	Variables need to be on same scale; clusters need to span similar size in space and be spherical	Translating health psychology into effective health communication: the american healthstyles audience segmentation project ^19^
**K-medoids** **(includes** **partitioning** **around** **medoids)**	Dissimilarity matrix	Data with outliers (more robust than k-means), mixed data that can be converted to Gower distance	Works with any distance matrix. Slightly more robust than k-means. Cluster centers are actual exemplars from data	Takes slightly longer to compute than k-means (trivial for most data sets)	Classification of incidence and prevalence of certain sexually transmitted infections by world regions ^20^
Distribution-based unsupervised clustering					
**Latent class** **analysis (type** **of finite mixture** **model)**	Categorical variables only	Data sets that are categorical or have been converted to categorical	One of few methods made to deal with categorical variables. Results simple to interpret (class probabilities). As mixture model, outputs metrics to evaluate optimal class number	Lose information as continuous distributions are discretized	Heterogeneity in urban park use of aging visitors: a latent class analysis ^21^
**Gaussian** **mixture models**	Multivariate Gaussian distributed data and therefore, continuous data only	Continuous data where it can be reasonably assumed subpopulations have Gaussian distributions on each variable (fits a distribution described by mean and standard deviation)	Where k-means assumes circular distribution, GMMs can fit any ellipse-shaped data	Inaccurate if continuous variables for a subgroup have non-normal distributions in some variables, which is often the case in real-world data	Consumer segmentation based on health-related motive orientations and fruit and vegetable consumption ^22^
**TwoStep** **clustering**	Continuous and categorical variables	Large data (solves some computation issues), hands-off solution. Similar to hierarchical clustering, but uses model-based estimate of distance. Sequentially groups records that are similar, then performs hierarchical clustering across these groups.	Provides list of relevance of each variable to clustering solution. Does the heavy lifting from start to finish, including optimal solution suggestion.	Order of records matters for final solution. SPSS only.	Subgrouping outpatients of an environmental medicine unit using SCL-90-R and cluster analysis ^23^
Supervised clustering					
**Decision tree** **(e.g. CHAID:** **chi-square** **automatic** **interaction** **detection)**	Any mix of variable types	Segmentations with one outcome variable to guide the process, and therefore the only supervised method in this table to produce segments as a side effect. Useful if segments should be optimally separated by one target variable.	Segment construction very transparent and splitting rules are explicit	Population split by most predictive variables only, so may not show much differentiation on other (otherwise actionable) variables. Requires relevant and reliably measured outcome. Leaves of tree should be profiled to enrich description beyond few variables in decision tree.	Financial profiling of public hospitals: an application by data mining ^24^
Manual (no algorithm)					
**Manually define** **cut-offs for** **groups (no** **clustering** **algorithm** **involved)**	Cut-offs determined in absence of data based on knowledge, or based on descriptive statistics of data	Situations in which there is strong knowledge about which variables are important, and common sense or ‘eyeballing’ can indicate what a relevant subgroup is (e.g. in ecommerce, everyone who hasn’t been on the website in 30+ days)	Fast, leverages domain knowledge, easy to implement, not subject to vagaries of algorithms	Will never be better than intuition, human mind can only consider handful of variables	Interventions targeting groups where segments are defined a priori, for example by location, age, literacy, or medical history ^25^

One consideration of algorithm choice is not analytic, but based on program constraints. For example, are implementers constrained to develop interventions for a limited number of segments? Should target segments represent at least a certain percentage of the market? Does the program care most about the size of a segment, or how much segment members are at risk of contracting or spreading a certain disease? Depending on these discussions (also the considerations on segment prioritization below), algorithm parameters – most notably the number of clusters requested from the algorithm – can be adjusted to satisfy these constraints.

A *priori*, it is not obvious which algorithm will yield the best solution of finding segments that are clearly distinct and pass the ‘sanity check’ of splitting the data in a way that makes real-world sense. Therefore, we recommend that researchers perform a set of iterative analyses with competing algorithms and compare them against the goals and constraints of the project. Overall, human judgment is crucial throughout analysis. Investing in good ways of communicating, visualizing, and comparing the different solutions with non-technical colleagues pays dividends when it is time to apply the segmentation solution. No part of the analysis should be isolated from the people making decisions on how segments will be used, and those with in-depth knowledge of the population.

**Figure 2.**
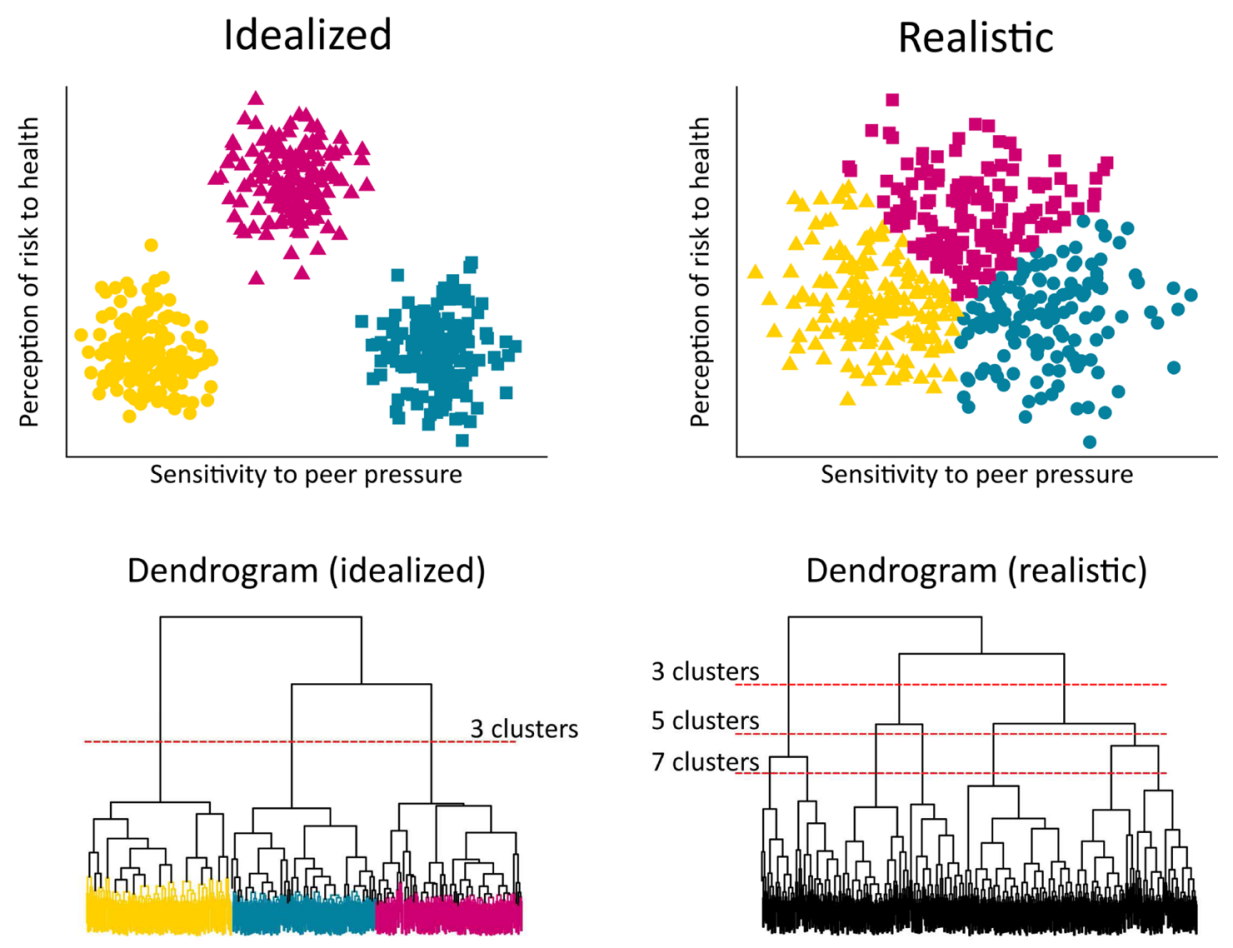
Idealized and realistic outputs of k-means (upper panel) and hierarchical (lower panel) cluster analysis. The reality of psycho-behavioral segmentation is that there are rarely perfectly-defined clusters. We illustrate this with simulated data showing a segmentation with two input variables in the context of getting a test for sexually-transmitted diseases (STDs): sensitivity to peer pressure to get the test, and perception of risk to health of STDs. In the idealized case (left side), there are clearly three clusters detected by the k-means algorithm, perfectly separated from one another. The dendrogram of these data in the bottom left also shows a clear point at which the tree can be 'cut' to define the clusters (as done in hierarchical clustering). In the real world (right side), however, variables are often normally distributed and noisy. K-means will still identify three segments because that number of segments was specified in advance, but clearly there is little actual ‘clustering’ of people. Similarly, the dendrogram of these data suggests potential cut-off points at several heights that seem equally reasonable. This is not to say clusters could not be useful in this case, as there are differences in sensitivity to peer pressure and risk perception between the segments. However, it could be reflected in tailored interventions that the individuals near the middle of the cloud are not all that different from one another. The code to generate these graphics is available at https://github.com/SurgoFoundation/segmentation.

The two clustering algorithms we focus on here - hierarchical clustering and k-means – are based on ‘distances’ between people, grouping people that are close together in a space created by the input variables. However, often data sets include categorical variables (such as male/female or urban/rural) that do not naturally create a space to calculate distances in. This requires the use of alternative algorithms. In other cases, the clusters have unusual shapes that are poorly handled by some algorithms but not others. To deal with these challenges and others, many types of cluster algorithms and distance metrics exist, a sample of which is described in Table 2. It is important to note that only rarely do real-world data conform to idealized examples, and only visualization and iteration will reveal the best way of generating segments (illustrated in Figure 2, code available as extended data ^17^).

***Hierarchical clustering.*** Hierarchical clustering starts by treating all data points as a single cluster and gradually splitting them up (‘divisive’), or treating each data point as its own cluster and gradually merging them (‘agglomerative’). This is often represented via ‘dendrograms’ showing the merging hierarchy visually ( Figure 2, lower panel). Even if it may not turn out to be a suitable end solution for a particular data set, we recommend using hierarchical clustering for initial visual exploration of the data. A key advantage of hierarchical clustering is that it can be used with continuous, categorical, or even ordinal and mixed data, depending on the distance measure used. Another advantage of this method is that it does not pre-define the number of clusters that a solution will be split into. It also shows a hierarchy of groupings, rather than a single grouping as k-means and similar algorithms do, making it well-suited to initial exploration of data. A disadvantage is that once merged into a segment, data points will remain allocated to that segment throughout the hierarchy, so once-constructed segments are not improved iteratively.

To carry out hierarchical clustering, analysts must first determine a distance measure between data points. Clusters are split or merged depending on whether data points are close to each other or far apart in a space determined by the input variables. Therefore, the distance measure chosen is important and determines the shape of the clusters. Euclidean distance, essentially a straight line between data points, is often a default for continuous variables provided in many analytical packages. For categorical or mixed data, other distance measures such as Gower distance, which relies on a mixture of distance measures as appropriate for each variable type, should be selected. Another metric of similarity only used for continuous data is the correlation between answers of any two individuals: a high correlation suggests the *pattern* of responses is similar (even if absolute values of responses might not be). Next, a measure of distance between clusters needs to be defined. Should clusters be merged based on the maximum (‘complete linkage’), minimum (‘single linkage’), or another (e.g. ‘average’) measure of distance between elements of each cluster? Complete linkage is often the default, such as in the ‘ hclust’ function in the R programming language, and different linkage methods change the resulting splits in the dendrogram. Analysts can plot the dendrograms and assess whether the resulting splits make sense.

***K-means clustering.*** The most commonly used segmentation algorithm is k-means clustering. It works only if data are continuous (or approximating continuity). It also requires that the number of clusters is set in advance. One key advantage of k-means is that it is relatively fast and straightforward, as fewer decisions must be made by the analyst. Another is the iterative improvement of clustering solutions: the algorithm first picks central points (‘centroids’) for the given number of clusters at random, allocates data points to their nearest centroid, and then averages across data points to compute new centroids. This process is repeated until a stopping criterion is reached, such as a set number of iterations. In practice, 20–50 iterations have been shown to be sufficient ^26^. Disadvantages of k-means clustering are that the number of clusters must be set beforehand; that, unlike in hierarchical clustering, clusters are designed to consist of a center with a sphere around it and so may not detect groups of different shapes ^27^ or of different sizes of spheres; and that it is not appropriate for categorical data (but modified versions of k-means are, see below).

***Other algorithm types and modifications.*** Depending on the types of variables and success of previous steps, other algorithms can be explored ( Table 2). Most commonly, practitioners will run into the issue of dealing with categorical data not amenable to k-means. This can be overcome by using a distance metric that allows for categorical variables (e.g. Gower distance) and feeding the distances into hierarchical clustering or partitioning around medoids (PAM). Alternatively, some algorithms, such as modified k-means algorithms for categorical data ^28^ or two-step clustering ^29^, can be used to deal with categorical data. Other approaches are conceptually more removed from the techniques introduced here, such as latent class analysis, in which a model of hidden classes is built and data points are assigned a likelihood of belonging to a class ^10^. This technique is particularly useful if the data consists only of categorical variables.

**Figure 3.**
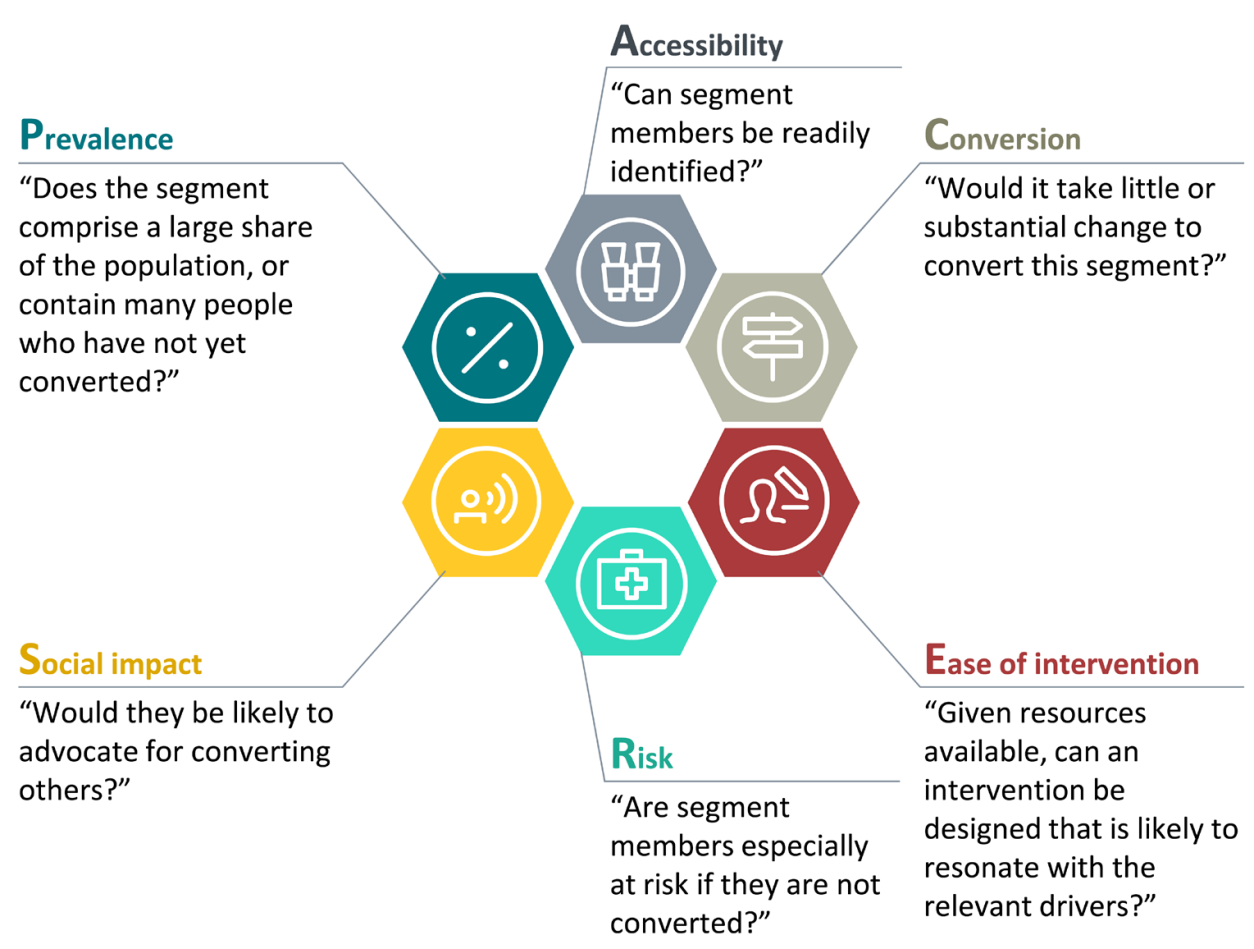
The PACERS framework for segment prioritization.

***Evaluating solutions.*** Evaluating competing solutions and determining the best number of clusters is once again a team effort. There is no unambiguously optimal number of segments, but in general a result is useful if segments are:

- distinct along relevant dimensions, with statistically meaningful differences between segments;
- actionable by the program (see Figure 3 for criteria programs can use to prioritize among segments).

The best solution will be the one that yields the most useful segments, and the merits of multiple solutions need to be understood before deciding on the optimal cluster number. This will involve generating multiple solutions and discussing with stakeholders the advantages and disadvantages of each. Apart from practical considerations, such as the number of targeted interventions or products that can reasonably be designed, various methods help decide on the number of clusters to use. No method completely avoids subjectivity, but the following steps combine a mix of methods to evaluate cluster solution outputs.

First, cluster identity can be plotted onto scatterplots of the data for different solutions to visually determine if obvious patterns are missed, or conversely whether patterns are forced onto the data even though it looks homogeneous (as is the case in Figure 2, right panel). In addition, analysts can deploy a mix of visual and statistical tools to evaluate separations. One example is the visual ‘Elbow’ method, where the overall within-cluster sum of squares is plotted for an increasing number of clusters. A ‘bend’ or ‘Elbow’ within the plot and a subsequent plateau indicate that adding further clusters will not explain much more of the variance. An alternative is the similar ‘silhouette’ method, looking at how well each data point fits into its cluster. Statistical indicators such as the Pseudo-F, Pseudo-T, or the ‘gap statistic’ can add insight. The Pseudo-F statistic gives the ratio of between-cluster variance to the variance within clusters. When the index is plotted against the number of clusters, large values indicate dense and distinct clusters. The Pseudo-T statistic is a measure of the difference between clusters merged together. Jumps in the index plotted against the number of clusters therefore point to an optimal number of clusters. The gap statistic tests within-cluster variance for a number of clusters and compares it to the null hypothesis of a random uniform distribution. In a plot of the output for different numbers of clusters, the optimum number of clusters maximizes the gap statistic. If the gap statistic suggests an unwieldy number of clusters, the number of clusters after which the slope of the increase starts to plateau is commonly selected ^30^.

A lack of ‘elbow’, or little variation in the statistical measures for different cluster solutions, may indicate that the algorithm used is unsuccessful at finding clusters, or that there are no clear clusters in the data. This does not mean there are no useful subgroups to be found, but the borders between the subgroups will be blurred (compare Figure 2, left panel versus right panel). In any case, analysts can back-check the solution by selecting a few segment members at random and looking at their input data: overall, do their responses justify segment membership? If not, another solution can be tried. Iteration is helpful in any case, and so the steps above can be repeated with different algorithms, including using methods in sequence. As in Sgaier *et al*. ^3^, hierarchical clustering can for example first be used to visually explore which number of segments makes the most sense for the data, followed by k-means clustering to refine the segments beyond the rigidity of the hierarchical approach. Less commonly, this order can also be reversed ^27^.

Another useful indicator to evaluate a cluster solution is to check whether available outcome measures that were not used to generate the clusters, but should correlate with behaviors of interest, are associated with clusters in expected ways. For example, in a cluster solution that looks at different health lifestyles, membership of a ‘health-oriented’ cluster should predict high, not low, rates of seatbelt use. Demographic variables can act as a further sanity check: if seatbelt use rises with age, that variable should also follow a similar distribution in the health lifestyle clusters (i.e., more older people would be expected in more health-oriented clusters). As another example, interventions campaigns focusing on alcohol abuse in the US might use smoking as an outcome measure correlated with heavy drinking ^31, 32^.

After settling on a solution, segments can then be characterized by tabulating or graphing the variables they differ on. In addition, they can be described narratively. Naming the segments with descriptive segment names so that their key differences are readily apparent is important and brings them to life.

### Prioritizing and targeting

***PACERS: a prioritization framework.*** In any program, resources rarely allow for targeting all segments with equal effort at the same time. An equal focus may also not be the desired approach in all cases. Programs can prioritize by considering a mixture of factors, summarized in the PACERS framework for segment prioritization ( Figure 3):

- Prevalence of a segment in the total population, and/or prevalence of people in the segment not yet converted to the target behavior.
- Accessibility, or how easily members of a segment can be identified: for example, a segment may be characterized by high involvement in social community structures
- Conversion effort required: for example, creating awareness of where a procedure is available in one segment may be easier than changing deep-seated social norms in another
- Ease of intervention given the available budget, expertise, and other resources of the implementing organization
- Risk, to health or otherwise, that a segment would be exposed to if not prioritized
- Social impact potential, for example that members of certain segments might be more likely to become advocates in their communities.

***Targeted intervention development to address segment-specific barriers.*** Segmentation is useful to develop interventions to specifically target and fit each priority segment’s key drivers and barriers. Interventions can take many forms, including mass communication campaigns, financial incentives, face-to-face support, and improving infrastructure conditions. For example, if some women do not use certain temporary contraception because of a fear of becoming permanently infertile, program designers can emphasize a corresponding message to them, or suggest an alternative product that fits their needs better. For another segment, the key barrier might be a social norm of demonstrating fertility by having many children, in which case more sustained work to change social norms is necessary.

Segment members can be targeted individually, or by counting on them to self-select into macro-level interventions, such as communication campaigns addressing several drivers at once. In either case, messages and intervention aides should be pilot-tested before scaling up their use.

**Figure 4.**
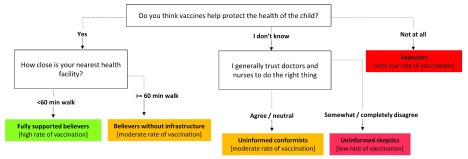
Representative schematic of a segment typing tool. For individual targeting, field workers or other stakeholders can use typing tools that quickly identify which segment an individual most likely belongs to. Splits in a decision tree-based typing tool can be based on categorical or continuous variables alike, and are chosen by the algorithm to identify members of each segment as accurately as possible. By giving responses to each question, a person is then allocated to a segment at the end of their path. Here, we show a hypothetical example of what a typing tool could look like to allocate a parent into existing segments relating to child vaccination behaviors. A parent in a given segment might be more or less likely to vaccinate their child, for different reasons. The field worker can then select an intervention or message that is most likely to resonate with that specific segment. For practicality, typing tools often stick to the three or four most predictive questions. However, that practicality has a tradeoff with typing accuracy: the more accurate a typing tool needs to be, the more questions must be asked.

One way of identifying segment membership for targeting individuals is to use a segment typing tool ( Figure 4). Such a tool takes only a few points of data and outputs the predicted segment of the individual. If the typing tool is deployed with pen-and-paper in the field, decision trees are a good option ^3^. Decision tree-based typing tools identify the most predictive questions to determine segment identity, constructing a series of questions to be asked in the field. If a computer is available when administering the typing tool, more computationally demanding typing tools, such as a discriminant analysis, can be used. Expected accuracy in the field can be determined by building the typing tool on a subset of the data (training set) and applying it to the remainder (test set). Often the typing tool may have trouble distinguishing between two segments, or perform poorly on one particular segment. A common decision point will be how much accuracy to sacrifice in the interest of keeping the typing tool short, and which segments are most critical to type accurately.

***Monitoring and evaluating the segment-based approach.*** **Monitoring** the segments over time helps identify which are growing, shrinking, or otherwise changing in relevance. For example, one segment might consist of pregnant women who live more than 5 km from a hospital. If one year later a government initiative builds many new facilities, this segment will shrink and become less relevant to invest in. Likewise, an excellent transportation system to facilities might be introduced, meaning the segment does not change in size (the distance to facilities is still the same), but it does not require further intervention as the underlying problem of access is solved. The size and relevance of each segment can be tracked with simple surveys or, in some cases, third-party data.

Impact evaluation of the segmentation approach can be done at several levels. First, key program performance indicators can be monitored to ensure that the segment-based approach is not compromising overall delivery of the program. Second, conversion or success rates of the program can be compared before and after introduction of the segment-based approach. One option is that the segment-based approach is rolled out in different places at different times using a “stepped wedge” approach. This is a pragmatic and efficient study design that allows for an estimation of the causal impact of the newly introduced method ^33^. Lastly, an impact evaluation can be performed for certain types of interventions, whereby the target individual gets typed and is then randomly given either an intervention that matches with their type, or a generic control intervention. Such a ‘generic’ intervention could be the one-size-fits-all intervention provided prior to introducing the segment-based approach, or a mismatched intervention. If the segment-matched individuals perform better on the target behavior than the control individuals, this provides causal evidence for the impact of the segment-based approach ^9^.

## Results

***Case study: segmenting men for HIV prevention.*** Voluntary medical male circumcision (VMMC) is a proven intervention for reducing men’s risk of acquiring HIV ^34^. Nevertheless, targets for VMMC uptake had plateaued in southern African countries, including Zimbabwe and Zambia. A variety of mostly qualitative studies from different geographies suggested diverse reasons for not undergoing the procedure, including fear of medical complications, social stigma, or perceived low risk of acquiring HIV ^35^. However, the relative importance of these heterogeneous barriers, as well as the possible existence of others, was unknown. To achieve a higher rate of VMMC, it was therefore necessary to quantitatively identify robust subgroups of men that differed on their barriers to VMMC, and target them with interventions addressing these barriers. This case study describes how a multi-stakeholder program deployed a segmentation project reflecting the end-to-end methods described above. The full approach, including the resulting segments and typing tool, are described elsewhere ^3^, but here we highlight several decisions made at key steps in the process framework introduced in Figure 1.

At the start of the project, the program leadership assembled a diverse team consisting of representatives of the Ministries of Health in Zimbabwe and Zambia, research agencies, non-governmental organizations (NGOs) with field expertise in delivering HIV programs in Zimbabwe and Zambia, and donors. Previous research identified the target group for VMMC interventions as 15–29-year-old males ^36, 37^, and a literature review identified possible drivers of VMMC ^3^. From existing data, it emerged as crucial to understand the stages men went through from awareness of VMMC to intention (or motivation) to undergo it, and finally to action. Qualitative journey mapping illuminated these stages, and qualitative decision-making games further explored potential barriers, filling a gap in existing evidence. The combined insights from reviewing the literature and novel qualitative research provided clear guidance on what questions to ask in the subsequent quantitative survey to obtain the most relevant and actionable variables for segmentation.

An experienced market research company was then hired to collect the survey data using face-to-face interviews and help determine appropriate sample size. The questionnaire was designed using continuous rating scales rather than categorical answers wherever possible, to enable greater flexibility for cluster analysis. Furthermore, each potential driver of interest was measured with multiple questions where possible to improve the accuracy with which the construct was measured (see Table 1). Field research staff entered responses into a tablet-based system, increasing data quality and completeness compared with pen-and-paper questionnaires.

The analytical process was iterative, with analysts regularly discussing results with the field and program teams as well as decision-makers. The approach taken embodies many of the steps in Figure 1. First, dimensionality reduction of the data ensured the cluster analysis was happening on relevant variables. Second, the team evaluated the solutions on actionability as well as statistical indicators, and labeled the segments based on the distribution of input variables as well as behavioral variables. Third, prioritization of segments aimed to ensure that emerging solutions were actionable in the most efficient way. Several of the segments showed barriers to segmentation that could be targeted, were sufficiently large to be relevant, and were at risk of HIV infection if left untreated. Finally, development of a typing tool allowed field workers to identify what segment a male belongs to using a pen-and-paper tool only, as opposed to a computational typing tool that would require an app and internet connection. Field workers were equipped with a set of targeted interventions, such as ‘pain-o-meters’ visualizing the discomfort that could be expected from the procedure for those segments especially fearful of pain ^5^. This segmentation solution is now being deployed at scale in both Zimbabwe and Zambia, and impact evaluations will be published at a later date.

It is informative to consider the drawbacks of a VMMC intervention deployed without knowledge of the segments in the population. Rather than segmentation, analysts might have focused on uncovering overall associations via predictive modeling. For example, a fear of pain and complications related to VMMC might be predictive of not being circumcised, prompting interventions that alleviate such fears. Multiple such predictors (e.g. shame, low risk perception) might be identified, and thus a non-segmented approach can also lead to a portfolio of interventions. However, the key drawback of this approach is that each intervention would have to be deployed to each man, even though most men will only have a subset of barriers to VMMC that need to be addressed. In other words, an approach not based on segments would have missed groups within the population that are characterized by specific barriers, missing the opportunity to react to such heterogeneity in the field. Another advantage of having access to the richness of segments is that it is easier to perceive the subtleties that differentiate sub-groups, rather than focusing solely on predictive relationships with behavior. For example, this can reveal media channels through which an intervention could be deployed to best reach a segment, or how several messages might be linked together.

## Discussion

This methods article outlies how decision-makers, program implementers and researchers can conceptualize, design, field, analyze, and act on a psycho-behavioral segmentation solution. Such a segmentation process is a team effort and should include all these groups to combine expertise in domain knowledge, behavioral research design, advanced data analysis, and intervention design. This approach, which provides a comprehensive overview of all the necessary steps and main decision points in one place, should make the process more accessible for both public-sector programs and private-sector companies, not least by enabling domain experts and data scientists to ask each other the right questions and zero in on the steps most relevant to them.

There are critical choices at most stages of the segmentation process ( Figure 1), and while we present the steps as a linear process for simplicity, iteration is in fact key, for instance between the stages of selecting variables and testing algorithms. Most importantly, segmentation follows the principle of ‘garbage in, garbage out’. When this happens, intervention selection and design is at risk of not matching the real drivers of behavior. Therefore, it is important to think carefully about the dimensions to segment on and how to obtain data that measure them. Algorithm choice and parameter selection is another crucial step, as no one algorithm will provide a clearly optimal solution without experimentation and iteration, involving both implementers and analysts ( Figure 2).

To supplement this article, we have selected additional practical resources to help practitioners apply segmentation in their programs ( Table 3).

**Table 3.** A selection of practical tools for cluster segmentation. This non-exhaustive selection provides a starting point to the practitioner to learn from case studies in the field, work through case examples of cluster segmentation using the R programming language, and refer to more detailed characterizations of the main clustering algorithms and their implementations in popular software packages.

Resource	Description
**Operational and case study resources** **from program practitioners**	
Customer segmentation toolkit ^40^	This manual by the Consultative Group to Assist the Poor (CGAP) focuses on planning and resourcing segmentation in low-resource settings, including case studies focused on financial service provision
Advanced audience segmentation for social and behavior change ^41^	Breakthrough ACTION, based at the Johns Hopkins Center for Communication Programs (CCP), give a brief overview of steps for successful segmentation programs and reference several case studies from programs in the field
**Practical use cases including datasets** **and/or code**	
Cluster analysis and segmentation ^42^	Sample data analysis use case used at INSEAD, using hierarchical vs. k-means clustering including a dataset and R code
K-means cluster analysis ^43^	Tutorial using k-means clustering in R
Unsupervised machine learning: The hclust, pvclust, cluster, mclust, and more ^44^	Overview of the main R packages for cluster analysis, with sample code
A quick tour of mclust ^45^	Overview and code examples of model-based clustering techniques for the R mclust package
**Selected overview resources and manuals** **for statistical analysis**	
The elements of statistical learning ^46^	Highly technical yet practical statistical textbook; section 14.3 discusses clustering in detail. Includes examples and links to key R packages
Survey of clustering data mining techniques ^47^	In-depth technical overview of major clustering algorithms and their parameters
Cluster analysis in STATA ^48^	Overview of clustering functionality in STATA, a popular statistical program
The SPSS TwoStep cluster component ^29^	IBM’s technical report on the TwoStep cluster algorithm for the SPSS software package
Cluster analysis ^49^	Extensive cluster analysis manual for the SYSTAT software package

Translating segment characteristics into actionable interventions that address the key drivers of their respective target behaviors requires deep knowledge of the intervention at hand, design thinking, and careful testing. Not all segments may need to be prioritized, and the PACERS framework we introduce here helps programs make those choices ( Figure 3). Finally, segments are never static in size and relevance, especially if interventions are deployed based on the segments. Successful interventions will shrink the most problematic segments and grow the thriving ones, and external forces may similarly affect the size and relevance of segments. Monitoring the segments ensures that the initial segmentation efforts pay off in the long term.

Cluster analysis, an unsupervised machine learning technique, is an excellent method for detecting patterns of similarity, in other words, for finding hidden groupings without having to state explicit assumptions on what these groupings should be. However, differences within populations can also be found using other methods ( Table 2). For instance, decision-tree analyses can hierarchically determine which variables and interactions statistically predict a target behavior from occurring. Unsupervised cluster algorithms are suitable if no assumptions about the groupings or data are made, and/or there are several outcomes of interest none of which should strongly drive the segmentation process. In contrast, decision tree-based methods are ‘supervised’, so can only classify individuals into groups that share as much as possible a single, pre-defined outcome of interest (such as ‘achieved a performance target or not’). They are most suitable when the main goal of an analysis is to find factors that strongly associate with a certain outcome ^38^. In this article, therefore, our focus lies on unsupervised learning because the main goal of segmentation is finding groups of people similar to each other, rather than finding variables that predict outcomes. For the same reason, we do recommend supervised methods to find the few most important factors that allocate individuals into known groups, as in a segment ‘typing tool’ ( Figure 4).

The approach described here has several limitations. First, segmentation is arguably as much art as science: it is not possible to recommend a single ‘optimal’ analytical approach, as algorithm selection and output interpretation both require experimentation and subjective judgment. However, this can be mitigated by experimenting with several algorithms and their parameters and transparently discussing the respective results. Second, this specific article, and the discussed study on voluntary medical male circumcision in Zimbabwe and Zambia, focuses on an input generation process that relies on primary field research. At the scale required for segmentation, this can be both expensive and time-consuming. In addition, segment definitions – though ideally stable – might change over time. A reliance on questionnaires also suffers from the usual concerns about social desirability and other respondent biases ^39^. Recently, psycho-behavioral segmentation relying on input data from passive social media data scraping has been shown to be effective at creating segments and targeting matching messaging to increase online purchasing ^9^. However, for many populations, such data profiles do not exist, and so must be generated through *de novo* research. The approach suggested here addresses both the (qualitative) depth and (quantitative) breadth required to generate meaningful psycho-behavioral data at scale, and researchers and implementers can pick and choose among appropriate approaches to data collection. Lastly, this article does not address in detail how to conduct full impact studies of the segmentation approach.

Applications for this approach are broad, and the ‘customer’ in customer segmentation can encompass such diverse groups as citizens interacting with a healthcare sector, professionals, and employees, as well as private-sector consumers of a specific service. Any focus area where customer behavior is currently sub-optimal, amenable to intervention, and likely to be influenced by different barriers and drivers is ripe for segmentation. There is no more excuse for one-size-fits-all solutions to multi-faceted problems.

## Data availability

### Source data

A full description of the case study results is presented in Sgaier SK *et al*. ^3^.

The data underlying this case report (anonymized survey responses) is owned by the governments of Zimbabwe and Zambia, and the authors have requested the respective governments to make the data publicly available. This request is currently subject to government approval. Until the data are publicly available, the data are made available upon reasonable request (criteria for access may apply subject to assessment by the respective governments). Requests for access to the data can be made to the following:

Zimbabwe

Ministry of Health and Child Care

Box CY1122, Causeway, Harare, Zimbabwe

Tel: +263 4 798555/60

Email: pr@mohcc.gov.zw

Zambia

Ministry of Community Development and Social Services, formerly Ministry of Community Development, Mother and Child Health

Community House, Sadzu Road, Lusaka, Zambia

Tel: +260 96 5792010

Email: info@mcdsw.gov.zm

### Underlying data

All data underlying the results are available as part of the article and no additional source data are required.

### Extended data

The code to generate the plots in Figure 2 as well as additional visualizations is available from the Surgo Foundation’s Github account: https://github.com/SurgoFoundation/segmentation

Zenodo: SurgoFoundation/segmentation: Segmentation code to support publication of Gates Open Research. http://doi.org/10.5281/zenodo.3249095 ^17^

License: MIT

R version: 3.6.0 beta (2019-04-11 r76379)

Packages:

ggplot2 (3.1.1)

mclust (5.4.3)

tibble (2.1.1)

dplyr (0.8.0.1)
