# Supplementary material for: Identifying population segments for effective intervention design and targeting using unsupervised machine learning: an end-to-end guide
Source: Gates Open Res. 2019 Oct 21;3:1503. Originally published 2019 Jul 2. [Version 2] doi: 10.12688/gatesopenres.13029.2 (PMC6820452; doi:10.12688/gatesopenres.13029.2)
Supplement: Peer review history

# Peer review: Reviewer response for version 2

**DOI:** 10.21956/gatesopenres.14221.r28098

**Published:** 2019-10-29

**Authors:** Michael D. Slater

**Affiliations:** 1 School of Communication, Ohio State University, Columbus, OH, USA

I have reviewed the revisions and found them satisfactory.

I confirm that I have read this submission and believe that I have an appropriate level of expertise to confirm that it is of an acceptable scientific standard.

# Peer review: Reviewer response for version 2

**DOI:** 10.21956/gatesopenres.14221.r28097

**Published:** 2019-10-21

**Authors:** Idil Cakim

**Affiliations:** 1 Nielsen, New York City, NY, USA

I've read the updated article and approve of the new version, which I find substantially more detailed.

I confirm that I have read this submission and believe that I have an appropriate level of expertise to confirm that it is of an acceptable scientific standard.

# Peer review: Reviewer response for version 1

**DOI:** 10.21956/gatesopenres.14139.r27493

**Published:** 2019-08-02

**Authors:** Idil Cakim

**Affiliations:** 1 Nielsen, New York City, NY, USA

The authors do an incredibly thorough job of explaining segmentation methodology, offering various statistical approaches and showing how machine learning still needs human guidance. In doing so, they reveal a practical and more precise way of identifying behavioral drivers and building personas. Their suggestion to customize communications and intervention plans according to segments is a valid one. They leave readers with a very flexible tool: the segmentation key, which can be easily adapted by field workers to identify patient types and implement interventions.

 As the authors note, the data behind the field work example they are citing is not yet fully public, but can be accessed upon request. This is the only reason I mentioned 'partly' replicable. But I would like to underscore that the method and the tools they are suggesting are clear and easy to adopt. One additional suggestion I would have is to devise an Appendix and expand on the case study by sharing the segments and the typing tool so it becomes more vivid for readers and offers them a prototype to follow. Future studies may involve a comparison of field work that was implemented based on segmentation vs not and compare compliance rates and public health results.

I confirm that I have read this submission and believe that I have an appropriate level of expertise to confirm that it is of an acceptable scientific standard.

# Response

**Published:** 2019-10-14

**Authors:** Sema Sgaier

**Response**

We thank the reviewer for their positive assessment of our article. We agree with the need to show segmentation results vividly. The case study has in fact been expanded on in an earlier publication, where the segments and the typing tool are shared in detail (Sgaier SK, Eletskaya M, Engl E, Mugurungi O, Tambatamba B, Ncube G, et al. A case study for a psychographic-behavioral segmentation approach for targeted demand generation in voluntary medical male circumcision. eLife. 2017;6.). We now refer more clearly to the detail this publication contains: The full approach, and including the resulting segments and typing tool, are described elsewhere [3], […]. We have also added a paragraph outlining what an intervention for VMMC may have looked like in the absence of segmentation (see response to Reviewer 1).

# Peer review: Reviewer response for version 1

**DOI:** 10.21956/gatesopenres.14139.r27490

**Published:** 2019-07-09

**Authors:** Michael D. Slater

**Affiliations:** 1 School of Communication, Ohio State University, Columbus, OH, USA

A good summary of multivariate classification (cluster-analytic) segmentation principles and practice, focusing on cluster-analytic approaches, which I agree are underutilized in health and other pro-social campaigns. I particularly like the heuristic suggestions regarding process and priorities, and the example provided of an intervention using these methods is an informative and appropriate one. This is a sophisticated and useful introduction to a topic that represents a complex blend of thoughtful qualitative decisions and intelligent and sophisticated use of quantitative data.

 The assessment of "partly" with respect to use of the method by readers is simply an acknowledgement that as with any statistical analysis, there is a bit to learn from manuals and even better from experienced practitioners. It would not be realistic to expect this article to comprehensively provide such instruction. I would suggest links to sites or texts providing additional guidance, perhaps for various widely used statistical packages.

 One recommendation that I suggest be added is use of outcome measures that are likely to be correlated with behaviors that are the focus of the effort to validate clusters (e.g. for an alcohol abuse campaign in the US, smoking can be used as it is correlated with heavy drinking). If the clusters perform at least as well as a series of demographic measures, they are a parsimonious method of clustering data (for an example, see Slater and Flora, 1991 ^1^, and discussion in Slater, 1996 ^2^).

 The assessment of only partial support for the claims based on the data presented is based on the fact that this is simply an initial description, without impact data yet available and without a treatment/control test of segmentation methods (an ideal) or at least a clear comparison of what the campaign might have looked like without the segmentation analysis compared with what was done, for a more qualitative and descriptive comparison. It seems to me that it is still incomplete as a case study without the impact data and some kind of post hoc assessment of ways in which the segmentation scheme seemed to work and/or not work.

 The assessment of only partial data availability is due to the fact that the data have not been approved for public posting by the client government agencies, though a method for requesting the data is provided.

I confirm that I have read this submission and believe that I have an appropriate level of expertise to confirm that it is of an acceptable scientific standard, however I have significant reservations, as outlined above.

## References

[1] : Health Lifestyles: Audience Segmentation Analysis for Public Health Interventions. *Health Education Quarterly*.1991;18(2) : 10.1177/109019819101800207 221-233 10.1177/109019819101800207 2055779

[2] : Theory and method in health audience segmentation. *J Health Commun*.1(3) : 10.1080/108107396128059 267-83 10.1080/108107396128059 10947364

# Response

**Published:** 2019-10-14

**Authors:** Sema Sgaier

**Response**

We thank the reviewer for the positive assessment and the insightful comments on our article. In our revised version, we now address the following points:

 

- *The assessment of "partly" with respect to use of the method by readers is simply an acknowledgement that as with any statistical analysis, there is a bit to learn from manuals and even better from experienced practitioners. It would not be realistic to expect this article to comprehensively provide such instruction. I would suggest links to sites or texts providing additional guidance, perhaps for various widely used statistical packages.*

 

We greatly appreciate the suggestion of adding resources from manuals and experienced practitioners, and now include a Table 3 containing additional practical guidance: operational and case study resources from program practitioners, examples of practical use cases including datasets and/or R code, and selected manuals and overview resources for statistical cluster analysis algorithms and packages. As the reviewer mentions, this is not an exhaustive list, but rather a narrow selection of tools we have found accessible and helpful to deepen the practical understanding required for segmentation.

**Resource and** **Description**

**Operational and case study resources from program practitioners**

**Customer segmentation toolkit** [1]

This manual by the Consultative Group to Assist the Poor (CGAP) focuses on planning and resourcing segmentation in low-resource settings, including case studies focused on financial service provision

**Advanced audience segmentation for social and behavior change** [2]

Breakthrough ACTION, based at the Johns Hopkins Center for Communication Programs (CCP), give a brief overview of steps for successful segmentation programs and reference several case studies from programs in the field

**Practical use cases including datasets and/or code**

**Cluster analysis and segmentation** [3]

Sample data analysis use case used at INSEAD, using hierarchical vs. k-means clustering including a dataset and R code

**K-means cluster analysis** [4]

Tutorial using k-means clustering in R

**Unsupervised machine learning: The hclust, pvclust, cluster, mclust, and more** [5]

Overview of the main R packages for cluster analysis, with sample code

**A quick tour of mclust** [6]

Overview and code examples of model-based clustering techniques for the R mclust package

**Selected overview resources and manuals for statistical analysis**

**The elements of statistical learning** [7]

Highly technical yet practical statistical textbook; section 14.3 discusses clustering in detail. Includes examples and links to key R packages

**Survey of clustering data mining techniques** [8]

In-depth technical overview of major clustering algorithms and their parameters

**Cluster analysis in STATA** [9]

Overview of clustering functionality in STATA, a popular statistical program

**The SPSS TwoStep cluster component** [10]

IBM’s technical report on the TwoStep cluster algorithm for the SPSS software package

**Cluster analysis** [11]

Extensive cluster analysis manual for the SYSTAT software package

 

- *One recommendation that I suggest be added is use of outcome measures that are likely to be correlated with behaviors that are the focus of the effort to validate clusters (e.g. for an alcohol abuse campaign in the US, smoking can be used as it is correlated with heavy drinking). If the clusters perform at least as well as a series of demographic measures, they are a parsimonious method of clustering data (for an example, see Slater and Flora, 1991, and discussion in Slater, 1996).*

We thank the reviewer for the helpful suggestion to use outcome measures that correlate with behaviors or concepts of interest to validate cluster solutions, and for the two references. We have now added this point to the section on ‘Evaluating solutions’ and cite the proposed references:

Another useful indicator to evaluate a cluster solution is to check whether available outcome measures that were not used to generate the clusters, but should correlate with behaviors of interest, are associated with clusters in expected ways. For example, in a cluster solution that looks at different health lifestyles, membership of a ‘health-oriented’ cluster should predict high, not low, rates of seatbelt use. Demographic variables can act as a further sanity check: if seatbelt use rises with age, that variable should also follow a similar distribution in the health lifestyle clusters (i.e., more older people would be expected in more health-oriented clusters). As another example, interventions campaigns focusing on alcohol abuse in the US might use smoking as an outcome measure correlated with heavy drinking [22, 23].

 

- *The assessment of only partial support for the claims based on the data presented is based on the fact that this is simply an initial description, without impact data yet available and without a treatment/control test of segmentation methods (an ideal) or at least a clear comparison of what the campaign might have looked like without the segmentation analysis compared with what was done, for a more qualitative and descriptive comparison. It seems to me that it is still incomplete as a case study without the impact data and some kind of post hoc assessment of ways in which the segmentation scheme seemed to work and/or not work. The assessment of only partial data availability is due to the fact that the data have not been approved for public posting by the client government agencies, though a method for requesting the data is provided.*

We agree that the case study we present in this protocol does not demonstrate segmentation impact in the sense of a controlled experiment. In a trial, for example, voluntary medical male circumcision rates could be compared in regions where segment-based versus one-size-fits-all interventions are deployed, or staged in a stepped-wedge design. Alternative approaches could be conducting pre-post segmentation assessments of target behaviors, or comparing the results of segment-based matched versus mismatched interventions, as in the Matz et al. (2017) reference we cite for online purchasing behaviors (although the latter would be ethically more questionable in a health context). Impact studies are extremely important, but are not the focus of this article, which aims to serve as a how-to guide. However, as suggested, at the end of our case study we have now added a section on what a campaign could have done without a segment-based approach:

It is informative to consider the drawbacks of a VMMC intervention deployed without knowledge of the segments in the population. Rather than segmentation, analysts might have focused on uncovering overall associations via predictive modeling. For example, a fear of pain and complications related to VMMC might be predictive of not being circumcised, prompting interventions that alleviate such fears. Multiple such predictors (e.g. shame, low risk perception) might be identified, and thus a non-segmented approach can also lead to a portfolio of interventions. However, the key drawback of this approach is that each intervention would have to be deployed to each man, even though most men will only have a subset of barriers to VMMC that need to be addressed. In other words, an approach not based on segments would have missed groups within the population that are characterized by specific barriers, missing the opportunity to react to such heterogeneity in the field. Another advantage of having access to the richness of segments is that it is easier to perceive the subtleties that differentiate sub-groups, rather than focusing solely on predictive relationships with behavior. For example, this can reveal media channels through which an intervention could be deployed to best reach a segment, or how several messages might be linked together.
